# Shared Genetic Architectures and Causal Associations Between Diabetic Retinopathy Progression and Frailty-Related Phenotypes

**DOI:** 10.3390/genes17060642

**Published:** 2026-05-31

**Authors:** Renxin Luo, Xiaotong Yu, Chen Huang, Shumei Tan, Yulin Tseng, Yue Feng, Xuemin Li

**Affiliations:** 1 Department of Ophthalmology, Peking University Third Hospital, Beijing 100191, China; 2 Center of Basic Medical Research, Institute of Medical Innovation and Research, Peking University Third Hospital, Beijing 100191, China

**Keywords:** diabetic retinopathy, frailty, genetic correlation, pleiotropy, shared genetic architecture

## Abstract

**Background/Objectives**: Observational studies have reported comorbidity between diabetic retinopathy (DR) and physical frailty, but their genetic interplay remains incompletely understood. This study evaluated shared genetic architecture and potential causal relationships between DR severity and frailty-related phenotypes (FRPs). **Methods**: GWAS summary statistics were analyzed for four DR phenotypes (broad DR, background DR [BDR], severe non-proliferative DR, and proliferative DR [PDR]) and six FRPs, including frailty index (FI), appendicular lean mass, handgrip strength (HGS), and walking pace (UWP). Global and local genetic correlations were estimated using LDSC, HDL, and LAVA. Causality was assessed using bidirectional Mendelian randomization (MR) and latent causal variable (LCV) analyses. Biological mechanisms were investigated using partitioned heritability, cross-trait meta-analysis, Bayesian colocalization, tissue and cell enrichment, prioritization (MAGMA/TWAS), and 3D chromatin annotation. **Results**: BDR and PDR showed positive genetic correlations with FI and negative correlations with UWP. Local genetic correlation analyses identified 82 significant regions, including signals on chromosome 6. MR supported a directional effect in which genetic liability to DR was associated with higher FI and lower HGS, with no evidence of reverse causation. LCV indicated partial genetic causality within a shared polygenic architecture. Cross-trait meta-analysis and colocalization highlighted the MHC region, prioritizing *C2*, *AIF1*, *NOTCH4*, and *EHMT2*. Additional non-MHC loci included the *BCL2L15* gene cluster and *TERF1*. **Conclusions**: DR and frailty share genetic determinants involving neurovascular, metabolic, and immune-inflammatory pathways, supporting an association between DR liability and frailty-related decline. Future longitudinal and functional studies are needed to validate these findings and assess candidate pleiotropic genes.

## 1. Introduction

Diabetic retinopathy (DR), a progressive microvascular complication of diabetes mellitus, has emerged as one of the leading causes of vision impairment and blindness worldwide [1]. Pathologically, the disease is associated with prolonged hyperglycemia, contributing to endothelial dysfunction, breakdown of the blood–retinal barrier, and ischemic damage [2]. DR typically progresses from asymptomatic nonproliferative stages, characterized by microaneurysms and retinal hemorrhages, to proliferative DR (PDR), characterized by pathological neovascularization and vitreous hemorrhage, potentially causing irreversible vision loss without timely intervention [3]. In 2020, DR affected 22.27% of adults with diabetes (over 103 million globally), with projections exceeding 160 million cases by 2045 as diabetes prevalence rises to 783.2 million [4,5]. In China, a recent national survey reported that the prevalence of DR and vision-threatening DR among adults with diabetes was 16.3% and 3.2% [6]. 

Frailty is a highly prevalent geriatric syndrome characterized by multisystem physiological decline and reduced resilience to internal and external stressors [7]. Closely intertwined with this syndrome is sarcopenia, a progressive generalized skeletal muscle disorder involving the loss of muscle mass and function, which shares significant overlapping features and pathophysiological mechanisms with frailty [8]. Consequently, quantitative traits such as the frailty index (FI) [9], appendicular lean mass (ALM) [10], hand grip strength (HGS) [11], and usual walking pace (UWP) are collectively recognized as key frailty-related or sarcopenia-related phenotypes that reflect the physical manifestation of this decline. Clinically, frailty and its related phenotypes are robust predictors of adverse outcomes, including falls, fractures, hospitalization, disability, and increased mortality [12]. 

Accumulating epidemiological evidence indicates a consistent, bidirectional comorbidity between diabetes-related complications and frailty. Frailty is markedly more prevalent in individuals with diabetes [13], with frailty/prefrailty independently predicting DR progression [14,15]. A recent Mendelian randomization (MR) study further suggests a causal relationship between standard DR and an elevated frailty index, suggesting that DR may be associated with frailty-related physical decline beyond its ocular manifestations [16]. Although the biological basis of this association remains unclear, genetic and mechanistic studies suggest that DR progression and frailty-related decline may partly involve overlapping vascular, metabolic, and immune-inflammatory pathways. Frailty GWASs have implicated neurological, cardiovascular, metabolic, and inflammation-related pathways. Studies of diabetic microvascular complications also support inherited susceptibility to DR and related vascular outcomes [9,17,18]. Mechanistically, chronic low-grade inflammation, endothelial dysfunction, oxidative stress, and vascular aging may promote retinal capillary injury and blood–retinal barrier disruption. These processes may also contribute to mitochondrial dysfunction, reduced physiological reserve, and frailty-related decline [1,2,7]. In DR, dysregulated VEGFA signaling may further increase vascular permeability and pathological neovascularization, supporting a vascular link between retinal microangiopathy and systemic vulnerability. However, the mechanisms and genetic architecture linking DR progression with frailty-related phenotypes remain insufficiently characterized.

This study utilized large-scale GWAS summary data to explore the shared genetic architectures and causal links between DR and frailty-related phenotypes (FRPs). Specifically, the analysis incorporated four DR phenotypes: broad DR, background diabetic retinopathy (BDR), severe non-proliferative DR (Severe NPDR), and proliferative DR (PDR). Additionally, six FRPs were included: FI, ALM, Left HGS, Right HGS, Low HGS, and UWP. Global genetic correlations were evaluated using Linkage Disequilibrium Score Regression (LDSC) and High-Definition Likelihood (HDL) methods. Bidirectional MR and the latent causal variable (LCV) method were applied to further explore potential causal associations. For significant global genetic correlation of the DR-FRP pair, subsequent analyses included local genetic correlation, partitioned heritability, cross-trait meta-analysis, Bayesian colocalization, functional annotation, gene prioritization, tissue and cell-type enrichment. 

## 2. Materials and Methods

**Figure 1:**
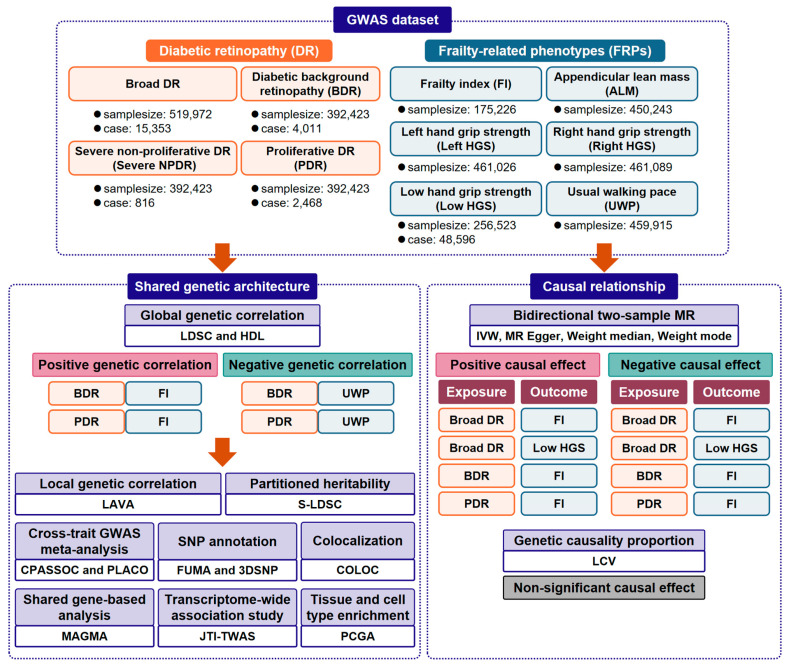
Overall study design of the shared genetic architecture between diabetic retinopathy and frailty-related phenotypes.

The overall study design and analytical workflow are summarized in Figure 1. Briefly, we first collected GWAS summary statistics for DR phenotypes and frailty-related phenotypes, followed by analyses of global and local genetic correlations. We then performed bidirectional MR and LCV analyses to evaluate potential causal relationships. For DR–FRP pairs showing significant global genetic correlations, downstream analyses were conducted, including partitioned heritability, cross-trait meta-analysis, Bayesian colocalization, functional annotation, gene prioritization, and tissue and cell-type enrichment. 

### 2.1. GWAS Data Sources

This study was based on publicly available summary statistics from GWAS and meta-analyses, all conducted in populations of European ancestry. 

GWAS summary statistics for the four specific DR phenotypes were obtained from the FinnGen consortium (https://www.finngen.fi/en, accessed on 10 September 2025). FinnGen is a large-scale research project that integrates genomic and health data from approximately 500,000 hospital- and population-based Finnish biobank participants (median age: 63 years; ~56.5% female). The DR phenotypes were identified using International Classification of Diseases-Revision 10 (ICD-10) criteria from the hospital discharge registry. The specific endpoints analyzed in this study were DR (DM_RETINOPATHY, R12, 15,353 cases), BDR (H7_RETINOPATHYDIAB_BKG, R9, 4011 cases), Severe NPDR (H7_RETINOPATHYDIAB_BKG_SEVERE, R9, 816 cases), and PDR (H7_RETINOPATHYDIAB_PROLIF, R9, 2468 cases). Endpoint-specific inclusion and exclusion criteria, case–control definitions, cohort construction, genotyping quality control, and imputation for FinnGen-derived DR GWAS summary statistics were performed according to standardized FinnGen protocols before public release. As this study used publicly available summary-level data, no additional individual-level inclusion, exclusion, or genotype quality-control procedures were performed by the authors.

The FI is a commonly used definition for frailty that is based on the accumulation of age-related deficits [9]. Summary data for the frailty index were retrieved from the most recent GWAS meta-analysis of the UK Biobank and Swedish TwinGene, including 175,226 individuals of European ancestry. In the source GWAS, FI was calculated by dividing the number of health deficits present by the total number of assessed deficits, yielding a continuous score ranging from 0 to 1, with higher values indicating greater frailty. The deficits covered multiple health domains, including symptoms, disabilities, and diagnosed diseases. Specifically, FI was constructed from 49 items in the UK Biobank and 44 items in Swedish TwinGene. In addition to FI, we included ALM, HGS, Low HGS, and UWP as complementary frailty- or sarcopenia-related phenotypes reflecting muscle mass, strength, and mobility. GWAS data for ALM (n = 450,243) [10], Left HGS (n = 461,026), Right HGS (n = 461,089), and UWP (n = 459,915) were sourced from the GWAS studies from the UK Biobank of participants aged between 48 and 73 years at recruitment. ALM was quantified by the sum of fat-free mass at the arms and legs measured by bioelectrical impedance analysis (BIA) using a Tanita BC418MA body composition analyzer (Tanita Corporation, Tokyo, Japan). The grip strength was obtained using a Jamar J00105 hydraulic hand dynamometer (Lafayette, IN, USA) that can be adjusted for hand size. UWP was defined as a category phenotype using the self-reported electronic questionnaire asking, ‘How would you describe your usual walking pace?’ Participants whose answers were ‘slow’, ‘steady/average’ or ‘brisk’ were classified as 0, 1, and 2, respectively, for analyses. 

Summary data for Low HGS (GCST90007526) were integrated from a meta-analysis of GWASs conducted in 22 independent cohorts, including the UK Biobank, the Health and Retirement Study in the United States, and the Framingham Heart Study [11]. The analysis involved a total of 256,523 individuals of European ancestry aged 60 years or older (48,596 cases and 207,927 controls) from the CHARGE consortium.

All original studies were approved by relevant ethics committees, and informed consent was obtained from all participants. As the present study was based solely on publicly available, de-identified GWAS summary statistics and did not involve individual-level identifiable information, no additional ethical approval was required. Further details on data sources are summarized in Supplementary Table S1 and the corresponding publications.

### 2.2. Global Genetic Correlation

The global genetic correlation between DRs and FRPs was estimated using two complementary approaches, LDSC [19] and HDL [20]. LDSC analysis was conducted with partial LD information from ~1.2 million HapMap3 SNVs using the 1000 Genomes European reference panel. For enhanced precision, the HDL method was employed, leveraging full LD information from a UK Biobank reference panel of 1,029,876 HapMap3 SNVs. Following multiple testing correction with the Benjamini–Hochberg (BH) procedure, a false discovery rate (FDR q-value) < 0.05 was considered statistically significant. Trait pairs exhibiting significant genetic correlations by both methods were designated as linkage pairs for subsequent analysis.

### 2.3. Local Genetic Correlation

Local genetic correlations were identified using the Local Analysis of [co]Variant Association (LAVA, version 0.1.5) tool [21]. For the analysis, the genome was divided into 2495 semi-independent LD blocks (average size ~1 Mb), defined by the 1000 Genomes European reference data. The process involved two stages: first, a univariate analysis identified blocks with significant SNP heritability for each trait (*p* < 0.05/2495). Bivariate analyses were then conducted exclusively on these pre-selected regions where both traits in a pair demonstrated significant heritability. A local genetic correlation was considered significant at a bivariate *p*-value < 0.05.

### 2.4. Two-Sample Mendelian Randomization Analysis

The two-sample MR analysis was performed to assess the potential causal relationship between DRs and FRPs. Instrumental variables (IVs) associated with each trait were selected using a genome-wide significance threshold of *p*-value < 5 × 10^−8^, and were subsequently pruned for LD at r^2^ < 0.001 within a 10,000 kb window, based on the European 1000 Genomes Project reference panel. 

The primary causal inference employed the inverse variance weighted (IVW) method, complemented by MR-Egger, weighted median, and weighted mode [22]. A comprehensive set of sensitivity analyses was conducted, including MR-Egger regression to detect directional pleiotropy, MR-PRESSO [23] to identify and correct outliers, and leave-one-out analysis to assess robustness. Heterogeneity was evaluated using Cochran’s Q test, and the Steiger test was used to confirm the assumed causal direction. 

A causal association was considered significant when the IVW estimate survived FDR correction using the Benjamini–Hochberg method (q-value < 0.05), showed consistent effect directions across all four MR methods, and passed sensitivity analyses. Associations with a nominal IVW *p*-value < 0.05 but an IVW FDR q-value ≥ 0.05 were considered potential causal associations.

### 2.5. Latent Causal Variable Analysis

LCV analysis [24] was employed to evaluate the genetic causality proportion (GCP) between DR progression and FRPs. This method uses LD scores consistent with the LDSC framework to construct a latent variable that mediates the genetic correlation between two traits. It then assesses whether this latent variable shows a stronger genetic association with one trait than with the other. The resulting GCP values range from −1 to 1; a high absolute value indicates partial genetic causality (where one trait drives the other), whereas a value near zero suggests horizontal pleiotropy. Significance testing was performed using *p*-values from the Z-score of the GCP. Multiple testing correction was applied using the BH FDR method. A strong causal signal was defined as an outcome satisfying both an FDR q-value < 0.05 and an absolute GCP exceeding 0.6 [25].

### 2.6. Partitioned Heritability

The genetic architecture shared between DRs and FRPs was explored by partitioning heritability using stratified-LDSC (S-LDSC) [26]. This approach assesses the contribution of 97 functional genomic categories, with annotations sourced from the ENCODE [27], Roadmap Epigenomics [28], and 1000 Genomes Phase 3 reference panels. S-LDSC quantifies the heritability enrichment within each category by computing LD scores for the constituent SNPs. The criteria for significant enrichment were an enrichment value > 1 and a corresponding *p*-value < 0.05.

### 2.7. Cross-Trait GWAS Meta-Analysis and SNP Annotation

To identify genetic variants shared between DRs and FRPs, a cross-trait GWAS meta-analysis was performed using two complementary methods: the Cross-Phenotype Association Test (CPASSOC) [29] and the Pleiotropic Analysis under Composite Null Hypothesis (PLACO) [30].

CPASSOC was applied to summary statistics to test for SNP associations, using its SHet statistic to account for potential heterogeneity in effect sizes. The significance criteria were P_SHet < 5 × 10^−8^, P_single_trait < 1 × 10^−3^ for each trait, and effect directions consistent with the sign of the global genetic correlation. PLACO was used to test a composite null hypothesis, generating a P_PLACO value from a multivariate model of shared SNPs. The threshold for pleiotropy was P_PLACO < 5 × 10^−8^, also requiring effect directions consistent with the sign of the global genetic correlation.

Variants that reached genome-wide significance in both analyses were advanced for functional characterization using the FUMA, version 1.6.3 [31] and 3DSNP V2.0 [32] web servers. The process involved defining independent significant SNPs (LD r^2^ < 0.6) and lead SNPs (LD r^2^ < 0.1), followed by the consolidation of loci within a 500 kb window, using the 1000 Genomes Phase 3 European panel for LD reference. A CADD score greater than 12.37 served as the cutoff to identify variants with potential deleterious effects.

### 2.8. Colocalization Analysis

A Bayesian colocalization analysis was performed using the “coloc” R package, version 5.2.3, in R software, version 4.5.1, to ascertain whether the lead SNPs associated with DRs and FRPs represented shared causal variants. This statistical framework computes posterior probabilities (PPs) for five mutually exclusive hypotheses at a given genomic locus: H0 (no association with either trait), H1/H2 (association with a single trait), H3 (association with both traits through distinct causal variants), and H4 (association with both traits mediated by a single shared causal variant). Summary-level data for variants within a ±100 kb window of each index pleiotropic SNP served as the input for this procedure. A PP for H4 (PP.H4) or H3 (PP.H3) greater than 0.70 was used as the threshold to support a shared causal variant or distinct causal variants, respectively.

### 2.9. Tissues and Cell Types’ SNP-Heritability Enrichment Analysis

To identify tissues and cell types exhibiting significant enrichment of SNP-based heritability for DRs and FRPs, the Phenotype–Cell–Gene Association (PCGA) analysis platform (https://pmglab.top/pcga, accessed on 10 September 2025) was utilized. This platform provides a comprehensive framework to jointly estimate trait-relevant tissues, cell types, and susceptibility genes using GWAS summary statistics as input. Its analytical power is derived from the integration of extensive bulk and single-cell transcriptomic data, which includes expression profiles from 54 human tissues (GTEx v8), 2214 human cell types, and 4384 mouse cell types. A statistically robust inference of phenotype–tissue and phenotype–cell type associations is achieved through an iterative estimation method, which is based on driver tissue estimation by selective expression. For this analysis, all 54 human tissues (GTEx v8) and cell types derived from the eye (n = 10), endothelial cells (n = 6), muscle tissue (n = 18), skeletal system (n = 25), brain (n = 227), and blood (n = 143) were selected.

Association test results were quantified as enrichment scores −log10(P), with a threshold of 1.301 serving as the marker for nominal significance (*p* < 0.05). Final determination of statistical significance considered on the BH method for FDR control, accepting only those associations with an adjusted *p*-value < 0.05.

### 2.10. Multi-Marker Analysis of GenoMic Annotation

A shared gene-based analysis using Multi-marker Analysis of GenoMic Annotation (MAGMA, version 1.10) [33] was employed to identify genes with pleiotropic effects on DRs and FRPs. By applying a multiple regression model, MAGMA accounts for the LD structure among SNPs to generate a single gene-level test of association. This procedure was run using its default settings, with genetic variants mapped to 20,259 protein-coding genes (Ensembl v110). Results associated with an FDR q-value < 0.05 were considered statistically significant.

### 2.11. Transcriptome-Wide Association Study

The transcriptome-wide association study (TWAS) was implemented using S-PrediXcan with joint-tissue imputation (JTI) models built from 49 GTEx v8 tissues [34]. This JTI approach refines gene expression prediction by leveraging shared regulatory patterns. Associations were considered statistically significant at an FDR q-value < 0.05. Pleiotropic gene expression was considered significant for genes showing FDR-corrected associations with both traits in a given linkage pair and tissue. The directionality of the reported z-scores classified these gene effects as risk-conferring (z > 0) or protective (z < 0). Notably, genes showing inverse z-score directions across different tissues were flagged as having bidirectional, tissue-specific effects regarding DRs and FRPs.

## 3. Results

### 3.1. Genetic Correlations Between Diabetic Retinopathy and Frailty-Related Phenotypes

**Figure 2:**
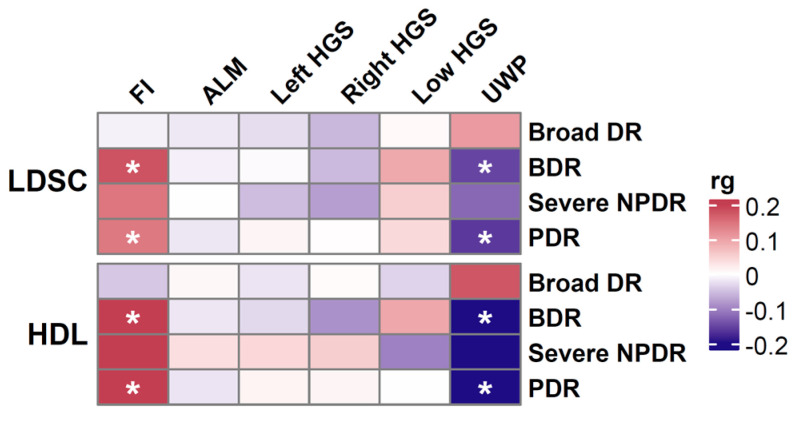
Global genetic correlations between diabetic retinopathy and frailty-related phenotypes estimated using LDSC and HDL. Asterisks indicate statistically significant genetic correlations after FDR correction (FDR q-value < 0.05). rg represents the genetic correlation coefficient.

Both LDSC and HDL analyses consistently identified FDR-corrected significant global genetic correlations between DRs and FRPs (Figure 2, Supplementary Tables S2 and S3). Specifically, both BDR and PDR showed positive genetic correlations with the FI and negative genetic correlations with UWP. The genetic covariance intercept between DRs and FRPs indicated no evidence of sample overlap bias (Supplementary Table S2).

**Figure 3:**
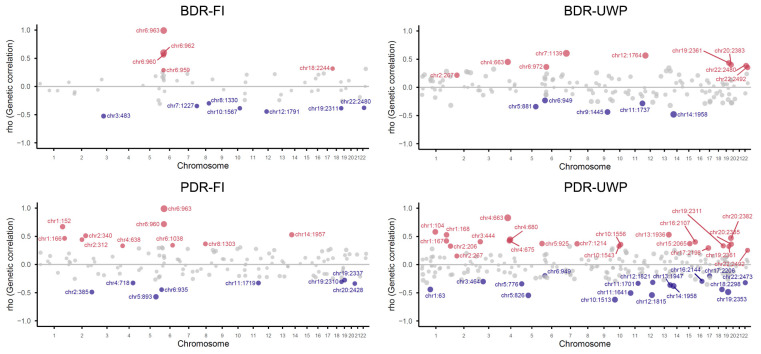
Local genetic correlations between diabetic retinopathy subtypes (background and proliferative) and specific frailty-related phenotypes (frailty index and usual walking pace) identified through LAVA method. Grey dots represent genomic regions without statistically significant local genetic correlations, while colored dots indicate significant local genetic correlations. Red dots indicate positive local genetic correlations, and blue dots indicate negative local genetic correlations.

Local genetic correlations are particularly advantageous for resolving genetic relationships that involve bidirectional effects. Using LAVA to characterize the genomic architecture behind the global correlations, our analysis revealed 82 significant local signals (*p* < 0.05) between DR subtypes (BDR and PDR) and the two FRPs (FI and UWP), which mapped to 73 distinct loci (Figure 3, Supplementary Table S4). For BDR, 12 and 14 significant loci were identified as correlated with the FI and UWP, respectively. For PDR, 18 loci were correlated with the FI, while 38 loci were correlated with UWP.

A substantial proportion of these local genetic correlations exhibited an effect direction concordant with their respective global genetic correlations (Supplementary Table S5). Among these concordant signals, four loci were identified across four trait pairs, suggesting them as potential pleiotropic hotspots. For both DR subtypes, loci 960 and 963 on chromosome 6 demonstrated highly significant positive correlations with the FI, whereas loci 1958 (chr14:23985937-24906056) and 949 (chr6:24950380-25684629) exhibited significant negative correlations with UWP.

### 3.2. Causal Relationship Between Diabetic Retinopathy and Frailty-Related Phenotypes

**Figure 4:**
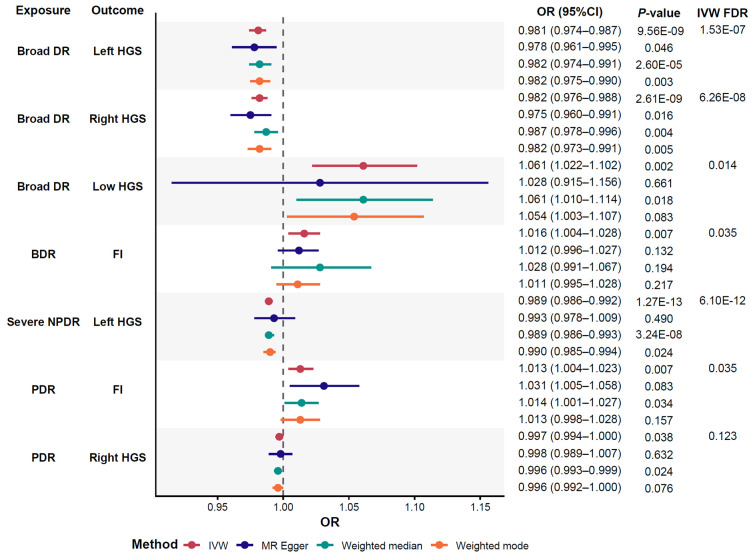
Forest plot of significant Mendelian randomization estimates for the effect of diabetic retinopathy (exposure) on frailty-related phenotypes (outcome).

To further and systematically assess potential causal relationships beyond the observed correlations, two-sample MR and LCV analyses were conducted on four DR phenotypes and six FRPs. In the two-sample MR analysis, no significant causal estimates were identified when the six FRPs were modeled as exposures and the four DR phenotypes as outcomes. Conversely, when assessing the potential effects of genetically predicted DR phenotypes on FRPs, significant associations were observed (Figure 4, Supplementary Table S6). Genetically predicted broad DR was significantly associated with adverse frailty outcomes, including elevated FI (βIVW = 0.0467) and increased susceptibility to Low HGS (βIVW = 0.0592), but was negatively associated with continuous HGS measures (Left: βIVW = −0.0193; Right: βIVW = −0.0186). Consistent with these findings, specific DR subtypes demonstrated similar causal patterns. Genetically predicted BDR and PDR were associated with increased FI (βIVW = 0.0159, βIVW = 0.0129, respectively). Furthermore, significant associations with reduced HGS were detected for Severe NPDR (Left: βIVW = −0.0112) and PDR (Right: βIVW = −0.0031).

In the LCV analysis, no trait pairs exhibited a strong causal signal based on the stringent criteria (FDR < 0.05 and |GCP| > 0.6) (Supplementary Table S7). While Severe NPDR showed a notable GCP of 0.4455 on FI (*p* = 0.0024), the FDR q-value (0.0576) was above the significance threshold.

Overall, two-sample MR provided genetic evidence supporting a potential directional association from DR liability to adverse frailty-related traits, including increased FI and reduced HGS, with no evidence of reverse causation. LCV did not identify strong genome-wide genetic causality under the stringent GCP criteria, suggesting that the DR–frailty relationship may involve partial causality within a broader shared polygenic architecture rather than a purely vertical causal pathway.

### 3.3. Partitioned Genetic Correlation Between Diabetic Retinopathy Subtypes and Specific Frailty-Related Phenotypes

Partitioned heritability analysis using S-LDSC identified significant enrichment of SNP heritability in distinct functional genomic annotations for the two DR subtypes and the two FRPs, highlighting both shared and trait-specific regulatory architectures (Supplementary Table S8). Among 97 functional categories, significant SNP enrichment was observed in 11 for BDR, 11 for PDR, 22 for FI, and 31 for UWP. Notably, five functional categories, including H3K27ac_HniszL2_0, SuperEnhancer_HniszL2_0, Backgrd_Selection_StatL2_0, CpG_Content_50kbL2_0, and MAF_Adj_ASMCL2_0, were significantly enriched across all four traits, suggesting shared regulatory mechanisms that contribute to the genetic correlation between DR and frailty.

### 3.4. Genomic Pleiotropic SNPs Shared for Diabetic Retinopathy Subtypes and Specific Frailty-Related Phenotypes

Cross-trait meta-analyses of the four DR-FRP trait pairs revealed 58 pleiotropic SNPs within 43 independent genomic loci showing significant associations (Supplementary Tables S9–S21). Of these loci, 39 were located on chromosome 6, three on chromosome 1, and one on chromosome 12, highlighting specific genomic hotspots. Further analysis demonstrated that these pleiotropic signals were concentrated in the Major Histocompatibility Complex (MHC) region on chromosome 6, which showed significant enrichment in associations with both FI and UWP across the two DR subtypes.

For DR-FI associations (Supplementary Table S21), a shared cluster of pleiotropic SNPs was identified across both DR subtypes, primarily enriched in MHC genes involved in antigen presentation, including HLA-DQA1 (rs9273068) and HLA-DQB2 (rs2857189), and inflammatory pathways like NCR3 (rs2736188). Colocalization analyses provided strong evidence for shared causal variants at rs114355928 in *C6orf10* (PP.H4 > 0.99), whereas other MHC signals (rs9273068, rs9405035) exhibited stronger evidence for distinct causal variants (PP.H3 > 0.70). Notably, rs9273068 exhibited a high CADD score of 12.19, suggesting potential deleterious functional impact. Complementing the MHC findings, the SNP rs1230682 on chromosome 1, located within *PHTF1* (a gene implicated in transcriptional regulation), was also found to be shared between both DR subtypes in relation to the FI. 

In the analyses with DR-UWP pairs (Supplementary Table S21), pleiotropy was again observed between both DR subtypes and this FRP, with significant enrichment of signals in the MHC region on chromosome 6. Nearby genes included those involved in immune complex assembly (*CFB*), heat shock protein response (*HSPA1A*), and histone clusters (*HIST1H2BF*, *HIST1H3D*). A notable variant, rs13198716 near *ABT1*, showed a high CADD score of 12.54, suggestive of functional impact. Colocalization analyses provided strong evidence for distinct causal variants at the MHC-associated signals rs1061783 (PP.H3 = 0.979) and rs3106190 (PP.H3 = 0.979). In addition, the SNP rs11102694 on chromosome 1, located within the AP4B1-AS1: *BCL2L15* gene cluster, which is known to play a role in modulating programmed cell death and gene regulation, was shared between both DR subtypes and UWP, supporting the pleiotropic signal observed on chromosome 1 for the FI.

### 3.5. Tissue and Cell Type Enrichment Between Diabetic Retinopathy Subtypes and Specific Frailty-Related Phenotypes

Tissue and cell type enrichment analyses revealed a complex shared genetic architecture between DR subtypes and FRPs (Supplementary Tables S22–S24). Specifically, BDR and PDR exhibited distinct systemic enrichment patterns with a shared emphasis on immune-related tissues. Significant heritability for BDR was concentrated in the spleen, whole blood, and small intestine, with cellular signals implicating specific immune subtypes such as T cells and macrophages. Similarly, PDR showed significant enrichment in the spleen and female reproductive tissues, although specific cell-type associations did not survive multiple testing corrections. In contrast, the genetic etiology of FRPs was predominantly driven by the central nervous system. Both FI and UWP showed significant enrichment in brain regions, including the cortex and basal ganglia. At the cellular level, this was mirrored by significant associations with neuronal subtypes for both phenotypes, with UWP displaying additional involvement of oligodendrocytes and specific immune lineages (NK and B cells).

Further analysis of cross-trait enrichment revealed specific overlapping mechanisms (Supplementary Table S24). For the BDR-FI pair, GTEx v8 signals were restricted to the uterus, with cellular overlap predominantly driven by broad immune lineages, including monocytes, macrophages, dendritic cells, and lymphocytes, as well as endothelial-related basal cells. The PDR-FI pair showed tissue enrichment in the tibial nerve and uterus, characterized by a more focused cellular overlap in M2 macrophages, specific dendritic cell subsets, and T/B lymphocytes. Regarding UWP-associated pairs, both BDR-UWP and PDR-UWP exhibited strong signals in cervical tissues (ecto- and endocervix). While BDR-UWP cross-enrichment was supported by basal cells and naive B cells, PDR-UWP displayed additional overlap in the tibial nerve but lacked significant concurrent enrichment at the specific cell-subtype level.

### 3.6. Genes Predicted Associations for Diabetic Retinopathy Subtypes and Specific Frailty-Related Phenotypes

To prioritize potential effector genes, MAGMA analysis first identified a substantial set of pleiotropic loci shared between DR subtypes and FRPs. Following FDR correction (q < 0.05), the number of significant genes ranged from 79 to 89 per trait pair (Supplementary Tables S25–S28). Subsequent JTI-TWAS analysis across 49 GTEx tissues revealed a broader range of tissue-specific associations, identifying between 87 and 111 significant gene-tissue combinations (Supplementary Tables S29–S32). 

**Figure 5:**
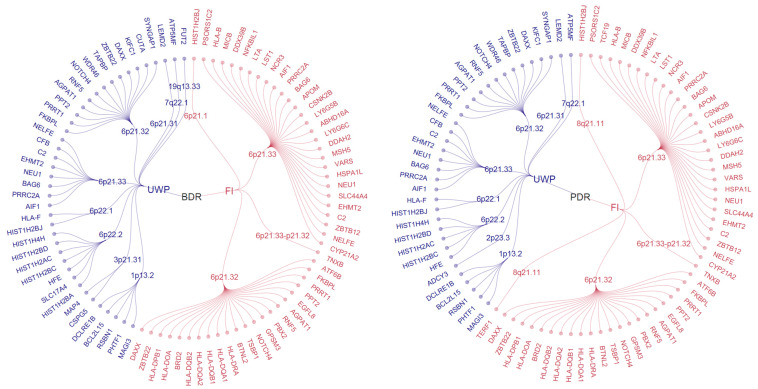
Circular dendrogram of the potential pleiotropic genes associated with diabetic retinopathy subtypes (background and proliferative) and specific frailty-related phenotypes (frailty index and usual walking pace).

To further identify the potential pleiotropic genes, the significant gene sets from both MAGMA and TWAS analyses were integrated with genomic annotations, including nearest genes and 3D chromatin interaction information obtained from the lead SNPs (Figure 5, Supplementary Table S33). Of the 78 unique pleiotropic genes identified, a core set of 16 was consistently associated across all four DR-FRP trait pairs. Notably, all 16 genes were mapped to chromosome 6, with all except the core histone gene HIST1H2BJ located within the MHC region, highlighting this locus as a hub of shared genetic effects.

Within this core gene set, distinct genetic effect patterns were observed, aligning with the genetic correlation directions of DR-FRP trait pairs. For the positively correlated FI, concordant effects were the most common pattern. Genes such as *AIF1*, *BAG6*, *C2*, *NELFE*, and *PRRC2A* exhibited Risk/Risk effects, whereas *DAXX*, *EHMT2*, *NOTCH4*, *PPT2*, and *RNF5* showed Protective/Protective effects. These genes were broadly enriched across immune, cardiovascular, metabolic, and neural tissues. Conversely, for the negatively correlated UWP, opposing effects were commonly observed. Genes such as *AIF1*, *BAG6*, *C2*, and *ZBTB22* showed a Risk/Protective profile, increasing DR risk but associated with a faster walking pace, while *DAXX*, *NOTCH4*, *PPT2*, and *RNF5* exhibited a Protective/Risk profile, reducing DR risk but linked to a slower walking pace. These pleiotropic effects were notably enriched in brain regions involved in motor and cognitive function, as well as in whole blood, adipose, and musculoskeletal tissues. Moreover, a few genes such as *AGPAT1* and *PRRT1* demonstrated bidirectional effects, suggesting complex, context-dependent regulatory roles.

In addition to the MHC locus, other chromosomal regions contributed to this shared architecture, with pleiotropic genes identified on chromosomes 1, 2, 3, 7, 8, and 19. For the PDR-FI trait pair, *TERF1* on chromosome 8 exhibited a concordant risk effect (Risk/Risk) in brain tissues and the pituitary. More complex patterns emerged for BDR-UWP and PDR-UWP trait pairs with a negative genetic correlation. Among the ten unique genes identified across these pairs, seven exhibited a Protective/Risk effect direction. This included a notable cluster of five genes (*BCL2L15*, *DCLRE1B*, *MAGI3*, *PHTF1*, and *RSBN1*) on chromosome 1, predicted to exhibit primary enrichment in arterial, neuronal, and metabolic tissues. This pattern was also observed in *FUT2* (chromosome 19) in secretory and gastrointestinal tissues and *ADCY3* (chromosome 2), specifically in nerve tissue. Furthermore, *ATP5MF* (chromosome 7) and *MAP4* (chromosome 3) showed concordant Protective/Protective effects, while *CSPG5* (chromosome 3) exhibited a Bidirectional/Bidirectional effect. 

Integration of functional genomic annotations with positional and 3D chromatin evidence further supports these genes as potential mediators of the shared genetic basis of DR and frailty. Key pleiotropic genes such as *C2*, *DAXX*, *EHMT2*, *NOTCH4*, *TNXB*, and *ZBTB22* were corroborated by both nearest-gene and 3D chromatin interaction evidence. Moreover, other notable genes including *AIF1*, *BAG6*, *NELFE*, *PRRC2A*, and *RNF5* were implicated through 3D chromatin interactions linked to high-frequency SNPs such as rs2857599, rs2243873, and rs2269426.

## 4. Discussion

This study provides the first systematic investigation of the shared genetic architecture and causal relationships between DR across varying severity levels and multiple FRPs, using large-scale GWAS summary statistics. Crucially, MR estimates provide evidence supporting a potential unidirectional causal effect, indicating that genetic liability to DR predisposes individuals to elevated FI and reduced HGS, whereas no evidence supports a reverse causal influence. Observational studies, including NHANES-based analyses [16], have reported comorbidity of visual impairment and frailty, but cannot readily distinguish shared biology from environmental or socioeconomic confounding. By integrating these quantitative genetic insights, the present analyses extend epidemiological observations toward molecular-level mechanisms. The findings indicate that comorbidity of DR and frailty reflects a shared genetic basis, with microvascular retinal pathology and systemic functional decline representing distinct clinical manifestations of interconnected immune, vascular, and neuro-metabolic pathways.

Bidirectional MR analyses suggest that genetic liability to DR is associated with poorer physical function, reflected by higher FI and lower HGS. These results are consistent with previous evidence linking retinal microvascular signs and systemic microvascular abnormalities to disability, functional loss, and poorer physical function in older adults [35,36]. Thus, retinal microvascular dysfunction may either contribute to functional decline or serve as a sensitive indicator of systemic physiological deficit accumulation. MR and LCV should therefore be interpreted as complementary rather than contradictory, because MR evaluates instrument-based directional associations, whereas LCV assesses the latent causal component of genome-wide genetic correlation. However, the lack of strong causality in the LCV analysis supports a more cautious interpretation: the link is unlikely to reflect a single, direct causal chain in which ocular pathology alone drives frailty. Instead, the pattern aligns with partial causality within a broader shared genetic architecture. DR progression may exacerbate decline through visual impairment and metabolic stress, while both DR and frailty largely arise in parallel from upstream genetic influences on vascular and metabolic aging.

Cross-trait meta-analyses and Bayesian colocalization identified the MHC region on chromosome 6 as a pleiotropic locus associated with the comorbidity of DR and FRPs. Strong colocalization evidence (PP.H4 > 0.99) at the *C6orf10* locus (rs114355928) substantiates the presence of shared causal variants. Although the function of *C6orf10* is not fully defined, its reported associations with multiple autoimmune and metabolic disorders, including rheumatoid arthritis and multiple sclerosis [37,38], support shared immune-related pathways involving immunosenescence and chronic inflammation. In this region, the complement gene *C2* showed pleiotropic effects, increasing risk for both DR and FI while exhibiting opposite-direction effects on walking pace, consistent with evidence linking complement activation to DR progression and choroidal neovascularization [39,40]. In addition, *AIF1*, *BAG6*, and *PRRC2A* were prioritized through 3D chromatin interactions with high-frequency variants. *AIF1*, involved in NF-κB signaling and macrophage activation [41,42], may contribute to systemic immune dysregulation relevant to retinal barrier impairment and musculoskeletal aging. In contrast, *NOTCH4* and *EHMT2* showed consistent protective effects across traits. *NOTCH4* is involved in endothelial cell fate decisions [43] and *EHMT2* encodes a histone methyltransferase [44], suggesting immune-epigenetic mechanisms that may protect microvascular and neuromuscular function. Collectively, the shared genetic architecture of DR and frailty is consistent with a pleiotropic immune-inflammatory module influencing both microvascular pathology and systemic functional decline.

Analyses beyond the MHC identified non-MHC pleiotropic clusters implicating neurovascular, apoptotic, and metabolic pathways. A locus on chromosome 1 (rs11102694 region) spanning *BCL2L15*, *PHTF1*, *DCLRE1B*, *MAGI3*, and *RSBN1* suggests shared mechanisms linking vascular and neural phenotypes. *BCL2L15* has been associated with apoptosis regulation and retinal tumor biology [45], and *DCLRE1B* has been reported as a hub gene for PDR progression [46], supporting the involvement of cell survival dysregulation in both retinal neurodegeneration and frailty-related neuromuscular decline. Along the PDR-FI axis, *TERF1* and metabolic loci including *ADCY3* and *FUT2* were prioritized. *TERF1* is linked to oxidative stress-related telomere damage and type 2 diabetes [47], consistent with evidence that telomere dysfunction contributes to aging phenotypes and that *TRF1* preservation mitigates neuromuscular decline [48,49]. *ADCY3* regulates hypothalamic energy homeostasis and is associated with obesity and insulin resistance [50], whereas *FUT2* influences gut microbiota composition, with *FUT2*-related dysbiosis potentially contributing to immune and neuroinflammatory modulation [51]. Although DR heritability is enriched in immune/endothelial cells and FRPs in CNS tissues [9,17], these loci collectively indicate convergence across arterial, neural, and metabolic tissues, involving apoptosis, telomere maintenance, and neuroendocrine regulation, which may contribute to parallel ocular and systemic degeneration.

Significant genetic correlations between BDR and FRPs suggest the potential value of considering systemic vulnerability even at earlier stages of DR [52,53]. The observed genetic overlap suggests that functional decline is not solely attributable to advanced visual impairment in late-stage PDR, but may arise during early retinopathy due to shared genetic susceptibility. These findings suggest that frailty-related phenotypes may be worth considering in future studies of systemic risk assessment among patients with early-stage DR. Prospective clinical studies are needed to determine whether incorporating frailty assessments into DR care can improve risk stratification or clinical management. Several pleiotropic genes may serve as potential therapeutic or biomarker candidates for this comorbidity. These include immune-related MHC-region signals encompassing *C2* and *AIF1* [54] and *NOTCH4*, which is involved in endothelial cell fate regulation [43]. Loci near the *BCL2L15* cluster and *TERF1* further point to pathways related to cellular stress responses and telomere biology [55].

Several limitations should be considered. First, most GWAS datasets included in this study were derived predominantly from individuals of European ancestry, which may limit the transferability of genetic correlations, causal estimates, and pleiotropic loci to non-European populations. Second, the use of summary-level data prevented individual-level analyses, detailed adjustment for clinical covariates, and evaluation of non-linear relationships that may influence both DR and frailty phenotypes. Third, UWP was derived from a self-reported questionnaire item rather than an objectively measured gait-speed test, which may introduce reporting bias or phenotype misclassification. Therefore, findings involving UWP should be interpreted with caution and warrant validation in future studies using objectively measured mobility phenotypes. Fourth, although multiple sensitivity analyses were performed, residual horizontal pleiotropy cannot be completely excluded and may still influence causal estimates. Nevertheless, the consistency across complementary methods, including global and local genetic correlation, bidirectional MR, LCV, and colocalization, supports the robustness of the shared genetic signals. In addition, our genetic analyses could not directly capture longitudinal clinical trajectories of DR progression and frailty-related decline. Future prospective cohort studies with repeated ophthalmic and frailty assessments are needed to clarify temporal dynamics and evaluate the clinical relevance of these genetic findings. These limitations should be considered when interpreting the broader applicability and potential clinical translation of our findings. In conclusion, this study characterizes the shared genetic architecture and potential causal links between DR and FRPs, implicating immune-inflammatory processes, neuroendocrine regulation, and neurovascular mechanisms in their comorbidity. The identification of pleiotropic genes, such as *C2* and *AIF1* involved in immune responses, *NOTCH4* implicated in endothelial cell fate regulation, and the *BCL2L15* cluster and *TERF1* related to apoptosis and telomere maintenance, further supports biological convergence between retinal microvascular pathology and systemic functional decline. These results suggest that DR may reflect broader aging-related physiological vulnerability rather than representing an isolated ocular condition. Future clinical studies should evaluate whether incorporating frailty assessments into DR care can improve risk stratification or clinical management. Cross-disciplinary care models integrating ophthalmology and geriatrics, together with longitudinal and multi-omic studies, are needed to clarify temporal relationships and underlying mechanisms and to inform future risk stratification and prevention strategies.

## Data Availability

The GWAS summary statistics analyzed in this study are publicly available from the original studies and public databases. GWAS summary statistics for diabetic retinopathy phenotypes were obtained from the FinnGen consortium (https://www.finngen.fi/en, accessed on 10 September 2025). Summary statistics for the frailty index were obtained from the GWAS meta-analysis of the UK Biobank and Swedish TwinGene. GWAS summary statistics for appendicular lean mass, left hand grip strength, right hand grip strength, and usual walking pace were obtained from GWAS studies based on the UK Biobank. Summary statistics for low hand grip strength are available under accession number GCST90007526. Further details on the data sources are provided in Supplementary Table S1 and the corresponding publications. No new individual-level genetic or phenotypic data were generated in this study.

## Supplementary Materials

The following supporting information can be downloaded at: https://www.mdpi.com/article/10.3390/genes17060642/s1, Table S1: Summary of GWAS datasets included in this genome-wide cross-trait analysis; Table S2: Genome-wide genetic correlations between diabetic retinopathy and frailty-related phenotypes identified by LDSC analysis; Table S3: Genome-wide genetic correlations between diabetic retinopathy and frailty-related phenotypes identified by HDL analysis; Table S4: Significant local genetic loci between diabetic retinopathy subtypes and frailty-related phenotypes identified by LAVA; Table S5: Significant local genetic loci with concordant effect directions to global genetic correlations between diabetic retinopathy subtypes and frailty-related phenotypes identified by integrating LAVA, LDSC, and HDL; Table S6: Results of two-sample Mendelian randomization analysis between diabetic retinopathy and frailty-related phenotypes; Table S7: Results of latent causal variable analysis between diabetic retinopathy and frailty-related phenotypes; Table S8: Significant partitioned SNP heritability in diabetic retinopathy subtypes and frailty-related phenotypes identified by stratified-LDSC; Table S9: Shared pleiotropic SNPs between background diabetic retinopathy and frailty index identified by CPASSOC; Table S10: Shared pleiotropic SNPs between background diabetic retinopathy and frailty index identified by PLACO; Table S11: Intersection of shared pleiotropic SNPs between background diabetic retinopathy and frailty index identified by CPASSOC and PLACO; Table S12: Shared pleiotropic SNPs between background diabetic retinopathy and usual walking pace identified by CPASSOC; Table S13: Shared pleiotropic SNPs between background diabetic retinopathy and usual walking pace identified by PLACO; Table S14: Intersection of shared pleiotropic SNPs between background diabetic retinopathy and usual walking pace identified by CPASSOC and PLACO; Table S15: Shared pleiotropic SNPs between proliferative diabetic retinopathy and frailty index identified by CPASSOC; Table S16: Shared pleiotropic SNPs between proliferative diabetic retinopathy and frailty index identified by PLACO; Table S17: Intersection of shared pleiotropic SNPs between proliferative diabetic retinopathy and frailty index identified by CPASSOC and PLACO; Table S18: Shared pleiotropic SNPs between proliferative diabetic retinopathy and usual walking pace identified by CPASSOC; Table S19: Shared pleiotropic SNPs between proliferative diabetic retinopathy and usual walking pace identified by PLACO; Table S20: Intersection of shared pleiotropic SNPs between proliferative diabetic retinopathy and usual walking pace identified by CPASSOC and PLACO; Table S21: Pleiotropic SNPs between diabetic retinopathy subtypes and frailty-related phenotypes; Table S22: Significant SNP-heritability enrichment results in 54 human tissues from GTEx v8 for diabetic retinopathy subtypes and frailty-related phenotypes analyzed by PCGA; Table S23: Significant SNP-heritability enrichment results in selected cell types from eye, brain, blood, muscle tissue, skeletal system, and endothelial cells for diabetic retinopathy subtypes and frailty-related phenotypes analyzed by PCGA; Table S24: Tissues and cell types cross-enriched in diabetic retinopathy subtypes and frailty-related phenotypes analyzed by PCGA; Table S25: Genes significant in both background diabetic retinopathy and frailty index in MAGMA analysis; Table S26: Genes significant in both background diabetic retinopathy and usual walking pace in MAGMA analysis; Table S27: Genes significant in both proliferative diabetic retinopathy and frailty index in MAGMA analysis; Table S28: Genes significant in both proliferative diabetic retinopathy and usual walking pace in MAGMA analysis; Table S29: Genes significant in both background diabetic retinopathy and frailty index in tissue-specific JTI-TWAS analysis; Table S30: Genes significant in both background diabetic retinopathy and usual walking pace in tissue-specific JTI-TWAS analysis; Table S31: Genes significant in both proliferative diabetic retinopathy and frailty index in tissue-specific JTI-TWAS analysis; Table S32: Genes significant in both proliferative diabetic retinopathy and usual walking pace in tissue-specific JTI-TWAS analysis; Table S33: Potential pleiotropic genes associated with diabetic retinopathy subtypes and frailty-related phenotypes.

## Abbreviations

The following abbreviations are used in this manuscript:

DR	Diabetic retinopathy
BDR	Background diabetic retinopathy
NPDR	Non-proliferative diabetic retinopathy
PDR	Proliferative diabetic retinopathy
FRPs	Frailty-related phenotypes
FI	Frailty index
ALM	Appendicular lean mass
HGS	Hand grip strength
UWP	Usual walking pace
GWAS	Genome-wide association study
ICD-10	International Classification of Diseases, 10th Revision
BIA	Bioelectrical impedance analysis
LD	Linkage disequilibrium
SNV	Single-nucleotide variant
SNP	Single-nucleotide polymorphism
LDSC	Linkage Disequilibrium Score Regression
HDL	High-Definition Likelihood
LAVA	Local Analysis of [co]Variant Association
MR	Mendelian randomization
IV	Instrumental variable
IVW	Inverse variance weighted
MR-PRESSO	Mendelian Randomization Pleiotropy RESidual Sum and Outlier
LCV	Latent causal variable
GCP	Genetic causality proportion
BH	Benjamini–Hochberg
FDR	False discovery rate
S-LDSC	Stratified Linkage Disequilibrium Score Regression
ENCODE	Encyclopedia of DNA Elements
CPASSOC	Cross-Phenotype Association Test
PLACO	Pleiotropic Analysis under Composite Null Hypothesis
FUMA	Functional Mapping and Annotation
CADD	Combined Annotation Dependent Depletion
PP	Posterior probability
PCGA	Phenotype-Cell-Gene Association
GTEx	Genotype-Tissue Expression
MAGMA	Multi-marker Analysis of GenoMic Annotation
TWAS	Transcriptome-wide association study
JTI	Joint-tissue imputation
MHC	Major Histocompatibility Complex
CNS	Central nervous system
NK	Natural killer
NF-κB	Nuclear factor kappa B
VEGFA	Vascular endothelial growth factor A
NHANES	National Health and Nutrition Examination Survey
CHARGE	Cohorts for Heart and Aging Research in
